# Personality, Foraging and Fitness Consequences in a Long Lived Seabird

**DOI:** 10.1371/journal.pone.0087269

**Published:** 2014-02-04

**Authors:** Samantha C. Patrick, Henri Weimerskirch

**Affiliations:** 1 Centre d’Etudes Biologiques de Chizé, CNRS-UPR1934, Villiers-en-Bois, France; 2 Biosciences, University of Gloucestershire, Cheltenham, United Kingdom; 3 Department of Zoology, University of Oxford, Oxford, United Kingdom; University of Debrecen, Hungary

## Abstract

While personality differences in animals are defined as consistent behavioural variation between individuals, the widely studied field of foraging specialisation in marine vertebrates has rarely been addressed within this framework. However there is much overlap between the two fields, both aiming to measure the causes and consequences of consistent individual behaviour. Here for the first time we use both a classic measure of personality, the response to a novel object, and an estimate of foraging strategy, derived from GPS data, to examine individual personality differences in black browed albatross and their consequences for fitness. First, we examine the repeatability of personality scores and link these to variation in foraging habitat. Bolder individuals forage nearer the colony, in shallower regions, whereas shyer birds travel further from the colony, and fed in deeper oceanic waters. Interestingly, neither personality score predicted a bird’s overlap with fisheries. Second, we show that both personality scores are correlated with fitness consequences, dependent on sex and year quality. Our data suggest that shyer males and bolder females have higher fitness, but the strength of this relationship depends on year quality. Females who forage further from the colony have higher breeding success in poor quality years, whereas males foraging close to the colony always have higher fitness. Together these results highlight the potential importance of personality variation in seabirds and that the fitness consequences of boldness and foraging strategy may be highly sex dependent.

## Introduction

The field of animal personalities has been one of fastest growing areas of behavioural ecology in the last decade. Early work sought to find concordance between the widely studied human personality framework (The big five) [1] and similar axes of behaviour in non-primates [1]. As a result much of what we know about personality variation comes from studies focussing on one of these five independent axes; most commonly the shy-bold continuum [1], [2]. This axis is particularly useful as it has repeatedly been shown to be heritable [3], and therefore has evolutionary potential, and tests can be standardised and compared across populations and species. However, more recently, work in the field of personality has evolved to include any measure of consistency between individual behaviour e.g. [4].

Concurrently, in the marine biology literature, there has been an increasing number of foraging studies on marine predators with the development of telemetry, and evidence is accumulating that there is a substantial individual component to foraging strategies, such as prey choice or spatial movement (Reviewed by [5]). However, while these individual differences in foraging can conceptually be considered as personality differences, the lack of overlap between behavioural ecology and marine biology has meant that there has been little attempt to implement the same analytical techniques, nor to consider these foraging behaviours within the framework of personality differences.

Seabirds, as top marine predators, offer an ideal system in which to consider the broad concept of personality differences because of the ease with which we can study their foraging behaviour at sea, and to carry out behavioural studies on land. Recently, there have been a few attempts to capture personality variation along the shy-bold continuum in seabirds. Patrick *et al*. [6] showed that wandering albatross (*Diomedea exulans*) show repeatable and heritable boldness and evidence from Black*-*tailed gulls *(Larus crassirostris)* showed that aggression at the nest, which is often considered to be part of a behavioural syndrome with boldness e.g. [7], [8], [9], was consistent between individuals [10], [11]. In addition, seabirds have become a model system to collect high resolution movement data, owing to newly developed miniaturised bio-logging technology which can most easily be deployed on these species [12]. Today individuals can be tracked across multiple trips and the repeatability of at-sea behaviour quantified for large number of individuals. As such the data exist to examine personality both by measuring consistency in boldness along the shy-bold continuum and consistency in foraging behaviour at sea. Finally, since seabirds are particularly long lived and are among the few animal populations for which long term demographic data exists [13], seabird models offer an unique opportunity not only to test the link between behavioural and foraging personalities, but also to estimate their consequences for fitness.

In this study we combine three data sets in an albatross species where we have a long term demographic data set, multiple at-sea foraging trips for individuals using telemetry, and recorded boldness of individuals at the nest. We measure individual boldness in black browed albatross, Kerguelen Islands, by recording the response to a novel object, a standard protocol used across many other taxa [1]. We also capture temporal and spatial aspects of foraging behaviour, using high resolution GPS loggers. Using the same methodology for both scores, we collapse each dataset into a single personality measure and consider the repeatability of these behaviours within the population. These analyses ask how much variation is explained by individuals demonstrating the same behaviour repeatedly and we show evidence that these two scores may represent a behavioural syndrome in seabirds. In the second part of this paper, we use these two measures of personality to explain variation in two important and widely measured aspects of at-sea behaviour allowing us to explain individual variation in these behaviours. We ask whether boldness predicts a) physical oceanographic habitat choice and whether boldness and foraging personality score predict b) overlap with human fisheries. Finally, we measure the reproductive success of individuals to determine whether either boldness or foraging personality score may correlate with fitness, both within a single year, and across reproductive attempts, when food availability may vary.

## Methods

Black-browed albatross (*Thalassarche melanophrys*) are large procellariform seabirds, which lay one egg per year, breed on sub-Antarctic islands and forage in the Southern Ocean [14]. They breed annually, show a reduced sexual dimorphism and can live for over 50 years [14]. This study was carried out at the Cañon des Sourcils Noirs study colony, Kerguelen Islands (48.4°S, 68.4°E) between 20^th^ December 2011 and 23^rd^ January 2012 (hereafter breeding season 2011), during late incubation and chick guarding. The study population was a sub-colony of 200 nests where a long term monitoring program started in 1979, with annual estimates of breeding success, recruitment and survival [15]. Both adults and chicks are caught and banded and in this study only reproductive data from 1988 onward were used as target birds did not breed before this date. Blood samples were collected for a subsample of 66 birds to determine the sex of individuals using standard protocols described elsewhere e.g. [16].

### 1) Personality Traits

We examined the behaviour of some individuals across two contexts (See table 1 for summary of study design):

**Table 1:** The structure of the analyses conducted in the study.

		“Boldness”	“Foraging personality score”
**1) Personality traits**			
Behaviour captured		Response to a novel object	Spatial and temporal foraging strategy
Score calculated using		PCA component 1	PCA Component 1
**2) Relationship with at sea behaviours**			
	a) Links to habitat choice		Analysis not possible as measures correlated
	b) Links to association with fisheries		b) Links to association with fisheries
**3) Fitness consequences**			
	a) Reproductive success 2011		a) Reproductive success 2011
	b) Reproductive success 1988–2011		b) Reproductive success 1988–2011

#### Data Collection: a) Boldness in response to a novel object (hereafter boldness)

The boldness of individual birds was tested on the nest by measuring the response to a novel object; a standard test for personality [2], [17]. A large pink volleyball (circumference = 59.5 cm), attached to the end of an 8 m carbon fibre fishing pole, was presented to each bird, immediately adjacent to the nest. The behaviour of the bird was filmed, using a GoPro video camera (Woodman Labs, Inc.) for one minute before the ball was removed. Each bird was exposed to the same test, with environmental variation minimised. A subsample of individuals were retested a minimum of seven days after the initial test to check for repeatability.

#### Data Collection: b) Foraging personality score

91 IgotU 120 GPS loggers (Mobile Action Technology) were deployed, where possible, on individuals of known boldness (N = 55). These devices, adapted with an 800 mAh battery, were programmed to record highly accurate locations every two minutes. Birds were caught on the nest and a device, previously waterproofed in heat shrink tubing, was attached to the back using TESA tape. The mass of the final package was 32 g, representing 1% of the adult body mass; well below the maximum 3% recommended [18]. GPS were left for multiple foraging trips wherever possible. In total we collected one trip for four individuals, two trips for 20 individuals, three trips for 23 individuals, four trips for 14 individuals, five trips for six individuals and six and seven trips for one individual. Three devices were not retrieved and four malfunctioned. 17 trips were excluded as they were recorded on late incubating birds (which did not hatch a chick) and exhibited much longer foraging trips compared to the others that were deployed after hatching during chick brooding (See electronic supplementary material, Appendix S1). Since foraging trips during incubation are generally longer than during brooding in Procellariiforms, for consistency we excluded the incubation tracks.

#### Analysis: a) Boldness

This test was based on a commonly used assay for boldness, defined along the shy-bold continuum, in response to a novel object. We selected this measure as it can be conducted at the nest and differences between tests can be minimised. Furthermore, similar tests can be carried out across populations and species, allowing comparisons to be drawn. Once chicks reached the age where they could defend themselves and begin to thermoregulate (ca. 11 days; [19]), parents no longer brood chicks continually and naturally spent more time standing than during incubation and chick brooding. For this reason we only used observations during which the bird was incubating or guarding a small chick (less than 11 days old), as standing was a component of our boldness test. However, it was not possible to age the individual chicks to the exact day, as we did not know the hatch date of offspring. The continual checking of adult birds to identify exact hatching date causes widespread disturbance in the colony, and so we used relative size to age chicks as less than or greater than 11 days. As such, we cannot fit chick age as a continuous variable in our models of boldness. As a result the final data set included 170 tests on 154 individuals, with 16 individuals (9%) tested twice. To minimise the effects of any variation in the approach of the ball, we excluded the first 30 seconds from each observation. Using the last 30 seconds for each test, we measured the number of times a bird “pecked” the ball (made contact), “lunged” - made a clear movement towards the ball (no contact), “vocalised” or “snapped” (opening and closing of the bill but not directed at ball), using JWatcher [20] (for ethogram see electronic supplementary material, Appendix S2)]. We also measured the duration of time (seconds) the bird spent sitting on the nest (rather than standing or raised on its tarsus). Principal component analyses (PCAs) are commonly used in personality research to collapse multiple scores into a small number of important, uncorrelated axes e.g. [21], [22]. They have been widely applied in personality research to group correlated behaviours into continuous personality scores and are particularly favoured as they avoid any subjectivity in grouping variables. We collapsed the recorded measures into a single score (principal component 1 (PC1); Table 2) and tested for sex differences (female: N = 21; male: N = 38) in PC1 by fitting sex as a fixed effect, with individual ID as a random effect. We estimate the repeatability in PC1 using mixed models to partition the variance explained by individual ID divided by the total population variance. A single “Boldness’ score for each individual was extracted from estimates from a general linear model (glm), including observation number (first or second observation of an individual), date and bird ID as fixed effects and the R package rptR [23] used to estimate repeatability.

**Table 2:** PCA output for boldness.

	PC1	PC2	PC3	PC4
Pecking	0.43	0.05	−0.70	0.34
Lunging	0.50	0.32	−0.17	−0.76
Vocalising	0.46	0.07	0.67	0.02
Snapping	−0.22	0.94	0.05	0.25
Sitting	−0.22	0.94	0.05	0.25
**Variance explained**	**0.32**	**0.20**	**0.19**	**0.15**
**Eigen values**	**1.60**	**0.98**	**0.96**	**0.77**

#### Analysis: b) Foraging personality score

We used a PCA to collapse four commonly used indices of foraging effort into a single score (Table 3; e.g. [5]): 1) Duration of trip (hours), 2) Maximum distance from the colony (Foraging range, km), 3) Maximum Latitude in a northerly direction,° 4) Maximum Latitude in a southerly direction, °. All points within 2 km of the colony were excluded to remove any effects of time at the nest. As GPS run continuously, without this buffer points when birds are at the nest would be included. This ensured we only considered the behaviour of the birds once they had left the colony. PC1 was extracted and we estimate the repeatability by using mixed models to partition the variance explained by individual ID divided by the total population variance in PC1. We tested for sex differences (female: N = 25; male: N = 48) in foraging personality score using linear models with PC1 as the response and sex as a fixed effect, and individual bird ID as a random effect. Final “foraging personality” scores per individual were calculated by extracting estimates from a glm, with PC1 as the response and bird ID as a fixed effect and the R package rptR used to estimate repeatability.

**Table 3:** PCA output for foraging personality score.

	PC1	PC2	PC3	PC4
Maximum Latitude in northerly direction	0.62	−0.33	0.18	−0.68
Maximum Latitude in southerly direction	0.17	−0.80	−0.36	0.45
Foraging Range	0.62	0.23	0.49	0.57
Duration forging trip	0.45	0.44	−0.77	0.00
**Variance explained**	**0.52**	**0.34**	**0.13**	**0.02**
**Eigen values**	**2.07**	**1.34**	**0.52**	**0.07**

#### Analysis: c) Correlation and association between personality traits

Behavioural syndromes occur when suites of personality traits are correlated within or between contexts e.g. [2]. Here we test for a correlation between boldness and foraging personality score using Spearman rank correlations (see [24]).

### 2) Link between Personality and Variation in Foraging Behaviours

We used the two personality measures extracted in section 1 to attempt to explain variation in widely reported aspects of at sea behaviour. First, studies have also suggested that seabirds differ in foraging habitat, which is linked with oceanographic features and often indicative of prey choice [25] but the causes of individual level variation are not fully resolved [5], [26]. Second, previous work has suggested that individuals differ in their association with fisheries, although whether these represent consistent strategies or opportunistic exploitation of resources is still unclear [26]–[29]. By testing the relationship with personality measures, we attempt to explain this individual level variation in these two classically reported marine behaviours.

#### Analysis: a) Foraging habitat

**Figure 1:**
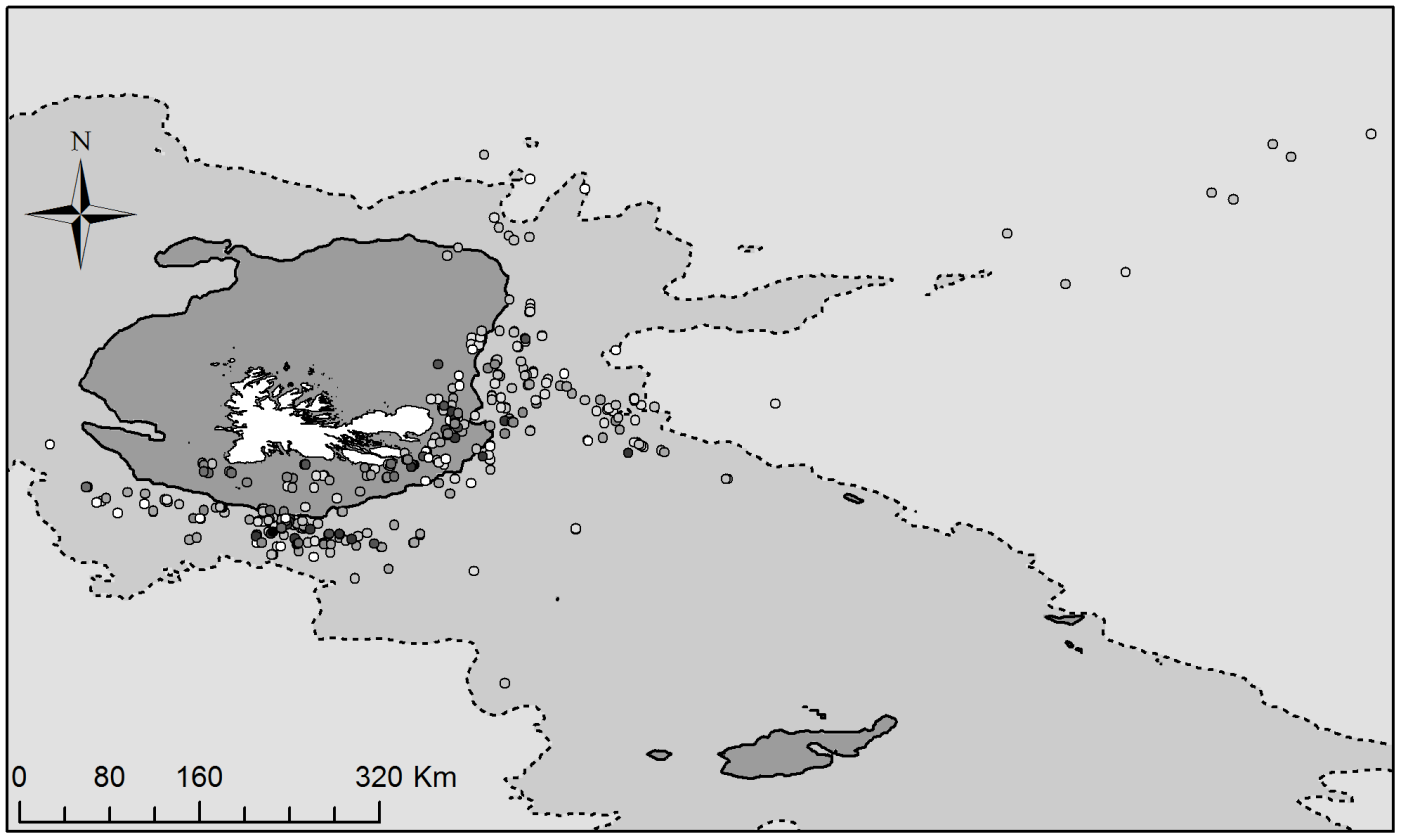
A map showing the relationship between boldness and all foraging areas in black browed albatross. Points show foraging zones for individuals, coded by the boldness and the average boldness of individuals foraging in these areas is plotted (0–200 m = Shelf, 200–2000 m = Shelf Edge and 2000 m+ = Oceanic). Boldness ranges from −3.95 (Shy; White) to 6.25 (Bold; Black), and as such, paler grey on the map shows shyer indiviudals forage here. Isobaths of 200 m (solid black line) and 2000 m (dashed black line) are shown. The breeding colony is shown by a star.

Given the huge distances albatross may cover in search of foods, there is high variation in the foraging conditions individuals experience. One major habitat feature in this population is the Kerguelen shelf which results in shallow waters around the colony, a high productive shelf edge up, and beyond deep oceanic waters (See Figure 1, Results). This species is known to specifically target the shelf edge [30] and while there is some covariance between distance from the colony and foraging habitat, the shelf edge begins at varying distances from the colony, with the closest point at 114 km and the furthest 703 km. The habitat birds forage in is likely to impact on prey type and quantity [25] and here we examine whether boldness correlates with an individual’s foraging habitat. As albatross often exhibit commuting phases during foraging trips [31], we isolate areas most likely to be associated with foraging by area restricted search (ARS). ARS is based on the First Passage Time method which identifies zones where individuals are expected to follow more sinuous paths while foraging and after successful capture of prey [32]. These changes in foraging behaviour can be identified from GPS tracks and used to locate active foraging zones. We excluded all data points when birds were on the water (speed <10 kmh^−1^) as these can lead to high levels of sinuosity, without any associated foraging behaviour. We used first passage time analysis (FPT; the time taken for a bird to cross a circle of given radius) as described by Fauchald and Tveraa [33] using functions and methods described by Pinaud [32] and developed using R [34]. Briefly, each track was interpolated at a scale of 1 km, and the FPT calculated every 1 km for a radius of 1 km–100 km. The logged variance in FPT was plotted against the radius to identify peaks in variance. For each peak in variance, the scale at which it occurred and the FPT threshold were extracted. All areas with a FPT greater than the threshold and more than 10 km apart were considered to be ARS zones (hereafter “foraging zones”). We extracted the foraging habitat at the central point of each ARS using standard marine habitats: ‘Shelf’ = depth less than 200 m; ‘Shelf edge’ = depth between 200 m and 2000 m; ‘Oceanic’ = depth greater than 2000 m. Using an ordinal regression, we fitted these three habitats with boldness, with sex as a fixed effect and trip ID, nested within, individual ID and as random factors.

#### Analysis: b) Interaction with fishing vessels

Albatross foraging behaviour can also be strongly influenced by human activity. However, while we know that some individuals forage at vessels, the causes and consequences of individual level variation are poorly resolved [26]–[29]. In the Kerguelen Exclusive Economic Zones, where all albatrosses were foraging (see results), French long liners were the only active vessels operating during the study period. This is an ideal situation whereby all fishing activity can be accounted for in analyses. Data on fisheries activity were made available from the Pechker data base, hosted at the Muséum National d’Histoire Naturelle in Paris [35]–[37] (Electronic supplementary material, Appendix S3). For the entire study period, the exact setting and hauling positions with times were available for all longlines. Lines are set at night, as one a series of measures to minimise the risk of accidental seabird bycatch [38] and boats return some hours later to haul the lines and remove fish from the hooks. This method of fishing has relatively little unwanted fish bycatch and so discarding during hauling is low [39]. Once the line has been completely retrieved, the vessels begin to move and discarding of offal and unwanted fish parts commences. Large aggregations of black browed albatross occur predominantly during these discarding periods, but can occur any point during fishing activity [40], [41].

The time and location for the end of hauling was used as the start of discarding. As discarding normally takes place within one hour from this point, we included a temporal buffer of plus one hour. As the maximum speed of a long liner is estimated to be approximately 19 kmh^−1^ when steaming, a spatial buffer of 19 km was created around the final hauling point to cover all potential discarding locations (hereafter “discarding zone”). For every GPS location we identified the presence/absence of any overlap with a discarding zone and this measure was fitted as the response variable, with a binomial error structure, with a) boldness b) foraging personality score, with sex as a fixed effect and individual trip, nested within bird ID, as random effects.

### 3) Fitness Implication of Personality Differences

#### Analysis: Fitness

For both personality scores, we measured the relationship with reproductive success, defined as a binary measure for fledging from all reproductive attempts (including attempts where egg did not hatch): 1 = chick survived to fledging (ringing age); 0 = chick did not survive to fledging for birds of known personality. We first used reproductive success in 2011, the year for which we collected foraging and boldness data, to examine the immediate implications of behaviour on fitness. Second, we used data from a long term database, examining the reproductive success of individuals for each attempt throughout the past 23 years (1988–2011). In all models we fitted a) boldness b) foraging personality score with sex as fixed effects and individual ID, and where appropriate, year, as a random effect. Both boldness and foraging personality score were mean centred to allow us to estimate the strength and direction of selection on the trait [42]. As there is evidence that the availability of food may determine the direction of selection acting on personality types e.g. [43], [44], we used oceanographic data to estimate prey abundance. In this population, the sea surface temperature anomaly (SSTa), which is the deviation from the average sea surface temperature (SST), has been found to be positively linked to reproductive success [45]–[47]. Specifically, it is the SSTa during September – November in the year of egg laying (Eggs laid in December) across the population range. We therefore extracted the SSTa values at a resolution of 0.5° between the maximum and minimum longitude and latitude of the population in 2011 (61°E –75°E and 45°S –57°S) and averaged across September, October and November, producing a mean SSTa for each year. This was then fitted as a covariate in models using long term estimates of reproductive success.

To test the significance of fixed effects, all models were run with and without the term of interest, fitted using Maximum likelihood (ML). All effects were tested by using ANOVA comparisons of full models to models without the term of interest. All analyses were carried out in R 2.15 [34] using packages ordinal and lme4 [48], Matlab 2009b and ArcGIS 10.

#### Ethic statement

All blood samples were collected using the minimum gauges needle, collecting only 0.2 ml of blood. There were no instances of continued bleeding, evidence of wound infection or response from the bird to the wound. Boldness tests were carried out for only one minute to ensure the disturbance to the colony was minimised. Each area of the colony was tested collectively to minimise the frequency of visits. One bird began to leave the nest during the personality observation and the test was immediately stopped and the bird returned quickly. GPS trackers weigh less than 1% of the mass of albatross and are highly streamlined and Tesa tape is used for attachment as it causes no lasting damage to the feathers (H. Weimerskirch, Pers. Obs). Breeding success of nests in the colony during the year of study was within the normal range and there was no evidence that the manipulations impacted on the colony. Licences and permissions were granted by the Ethic Committee of Institut Polaire Francais (IPEV) and by the Préfet of Terres australes et antarctiques francaises (TAAF) after advices from the Comité de l’Environnement Polaire (CEP).

## Results

### 1) Consistent Behavioural Differences

#### a) Boldness

**Figure 2:**
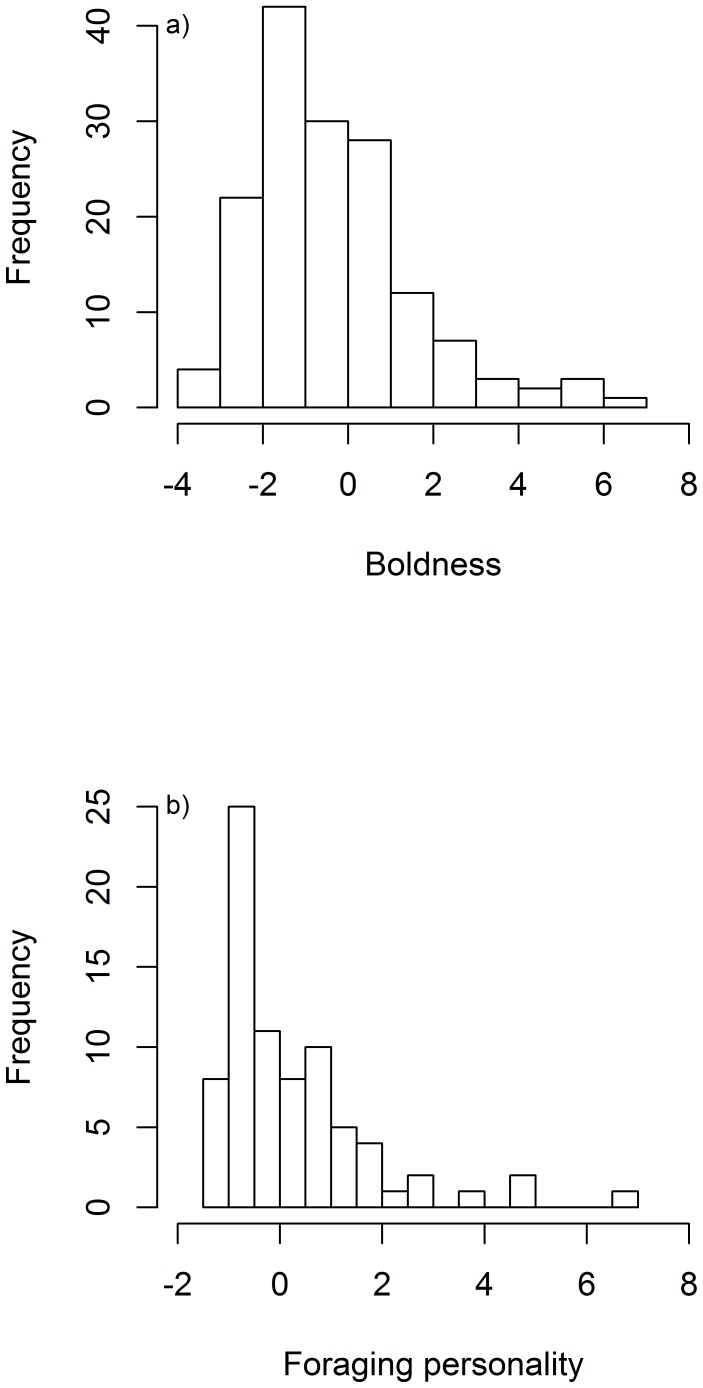
Histograms showing the frequency of personality types through the population. 2a: The frequency distribution of boldness scores among adult black browed albatross. 2b: The frequency distribution of foraging personality score among adult black browed albatross.

Principal component one explained 32% of the population variation in response to the novel object (Table 2), which is comparable to other studies using PCA to derive personality scores e.g. [21], [43]. This is interpreted as representing a measure of boldness when faced with a novel object. Observation number (χ^2^_1_ = 1.08; p = 0.30) and date (χ^2^_1_ = 3.24; p = 0.07) were included as fixed effects to account for variation between boldness tests. Although observation number was not significant, with the small number of repeats it is possible we did not have the power to detect such an effect, and so to be conservative we maintained it in the final model. Boldness scores ranged from −3.95 (shy individuals) to 6.25 (bold individuals), with a mean of −0.33±0.15 (Figure 2a). Individual albatross showed consistent boldness towards a novel object with a repeatability of 0.32±0.22. However, our low number of replicates gave insufficient power to demonstrate whether this was a significant repeatability (CI: 0.00–0.72; p = 1.00). There were no sex effects on boldness (χ^2^_1_ = 0.00; p = 0.96; Table 4).

**Table 4:** The main relationships between personality scores, foraging behaviours and fitness.

Response variable	Explanatory variable	Results		
*1a) Boldness*	Sex	χ^2^_1_ = 0.00	p = 0.96	N = 66
*1b) Foraging personality score*	Sex	**χ^2^_1_ = 27.99**	**p<0.001**	**N = 73**
*2a) Foraging Habitat*	Boldness	**χ^2^_1_ = 5.56**	**p = 0.018**	**N = 55**
	Sex	χ^2^_1_ = 2.88	p = 0.09	N = 78
	Boldness * Sex	χ^2^_1_ = 0.52	p = 0.47	N = 51
*2b) Fisheries overlap*	Boldness	χ^2^_1_ = 0.54	p = 0.46	N = 55
	Sex	χ^2^_1_ = 0.49	p = 0.48	N = 78
	Boldness * Sex	χ^2^_1_ = 0.00	p = 0.95	N = 51
	Foraging personality score	χ^2^_1_ = 0.07	p = 0.80	N = 78
	Sex	See above		
	Foraging personality score * Sex	χ^2^_1_ = 0.52	p = 0.47	N = 73
*3) Relationship with* *reproductive success 2011*	Boldness	χ^2^_1_ = 0.29	p = 0.59	N = 52
	Sex	NA		
	Boldness * Sex	χ^2^_1_ = 0.01	p = 0.92	N = 59
	Foraging personality score	*χ^2^_1_ = 3.06*	*p = 0.08*	*N = 78*
	Sex	See Above		
	Foraging personality score * Sex	χ^2^_1_ = 0.88	p = 0.35	N = 73
*3) Relationship with* *reproductive success (last 23 years)*	Boldness	NA		
	Sex	NA		
	Boldness * Sex	**χ^2^_4_ = 18.08**	**p = 0.001**	**N = 59**
	Foraging personality score	NA		
	Sex	NA		
	Foraging personality score * Sex	**χ^2^_4_ = 15.32**	**p = 0.004**	**N = 73**

Numbering of response variables links to those used in the methods and results sections. Bold results p<0.05; Italics p<0.1.

#### b) Foraging behavior

Principal component one explained 52% of the variance in foraging personality score (Table 3). Scores ranged from −1.96 to 6.73 (Figure 2b), where birds with a lower value foraged nearer the colony, made shorter trips, rarely travelling north from the colony. All components had a positive loading, although maximum latitude in a southerly direction had the weakest loading. The mean foraging personality score was 0.14±0.15. Birds were repeatable in their foraging personality score (r = 0.49±0.07; p<0.001) and there were strong differences between the sexes (χ^2^_1_ = 27.99; p<0.001; Table 4). Females showed a higher foraging personality score (1.07±0.20) than males (−0.40±0.15) showing that they made longer foraging trips, travelled further from the colony and were more likely to head in a northerly direction.

#### c) Correlation between two personality scores

There was a negative correlation between the two personality scores of −0.27 which was close to significant (p = 0.056), suggesting they show indications of a behavioural syndrome, with bolder birds making shorter trips away from the colony. With a larger sample size we would have the power to test whether these represent a syndrome and examine correlated selection acting on the traits. When looking within the sexes there was a negative correlation between the two traits but this was stronger in males (Males: r = −0.30; p = 0.12; Females: r = −0.13; p = 0.29), suggesting that with a larger sample size we could examine sex differences in syndromes.

### 2) Link between Personality and Variation in Foraging Behaviours

#### a) Foraging habitat

Bolder individuals were more likely to forage on shelf than the shelf edge, and least likely to foraging in oceanic areas (χ^2^_1_ = 5.56; p = 0.018; Table 4; Figure 1). There was no interaction between boldness and sex (χ^2^_1_ = 0.52; p = 0.47; Table 4), nor sex differences in foraging habitat (χ^2^_1_ = 2.88; p = 0.09; Table 4).

#### b) Association with fishing vessels

Out of 152 trips, 34 (22%) overlapped with fisheries, which represented 23 out of 49 birds (47%) which interacted with fisheries during at least one trip. However, for trips where birds interacted at least once with a vessel, the average proportion of time spent at fishing boats was 4.2% ±0.40. This represented a between 0.3–5.5 hours at vessels. Neither boldness (χ^2^_1_ = 0.54; p = 0.46; Table 4), sex (χ^2^_1_ = 0.49; p = 0.48; Table 4) nor the interaction (Boldness * sex: χ^2^_1_ = 0.00; p = 0.95; Table 4) influenced the overlap with fisheries. Neither foraging personality score (χ^2^_1_ = 0.07; p = 0.80; Table 4), nor the interaction (Foraging personality score * sex: χ^2^_1_ = 0.52; p = 0.47; Table 4) influenced the overlap with fisheries.

### 3) Fitness Implications

Boldness did not show any correlation with reproductive success in 2011 (χ^2^_1_ = 0.29; p = 0.59; Table 4), nor an interaction between boldness and sex (χ^2^_1_ = 0.01; p = 0.92; Table 4). As one would predict, there was no relationship between sex and reproductive success in 2011 (χ^2^_1_ = 0.97; p = 0.32; Table 4). Foraging personality score was also not linked with reproductive success in 2011 (χ^2^_1_ = 3.06; p = 0.08; Table 4), although there was a trend for birds with a lower foraging personality, which hence foraged nearer the colony and made shorter trips, having a higher reproductive success in this year. There was no interaction between foraging personality score and sex in 2011 (χ^2^_1_ = 0.88; p = 0.35; Table 4).

**Figure 3:**
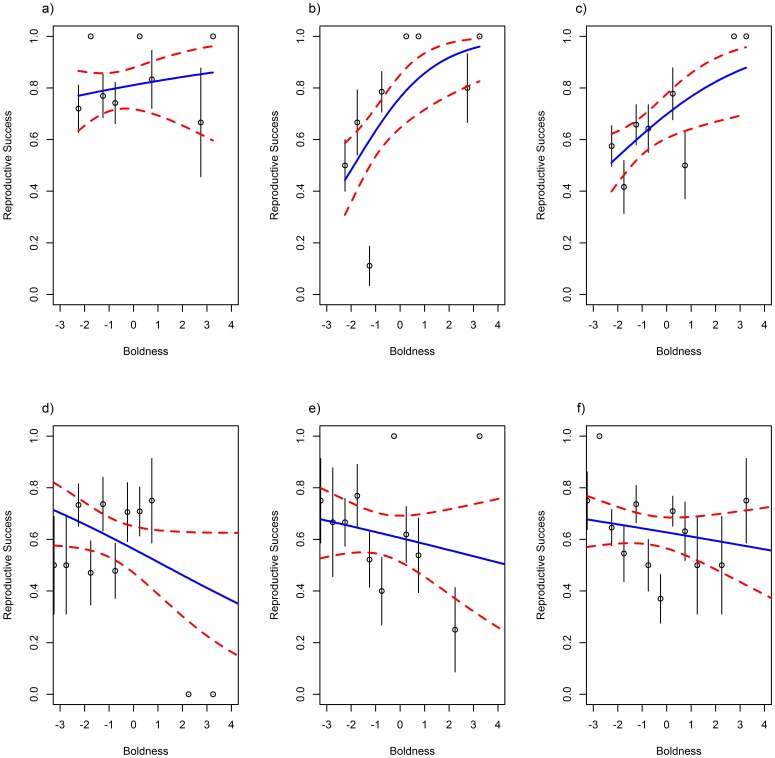
The relationship between boldness and reproductive success across the last 23 years. a) Females Low SSTa years: = −0.60< SSTa <−0.36; b) Females Medium SSTa years: −0.36< SSTa <−0.15; c) Females High SSTa years: −0.15< SSTa <0.13; d) Males Low SSTa; e) Males Medium SSTa; f) Males High SSTa. Boldness, while being a continuous measure, is grouped here for plotting purposes only. As the raw data is formed of zeros and ones, plotting grouped means provides a much more informative plot. Points represent group means, with standard error bars. Model predictions are plotted in a solid line with 95% confidence intervals in dashed lines.

**Figure 4:**
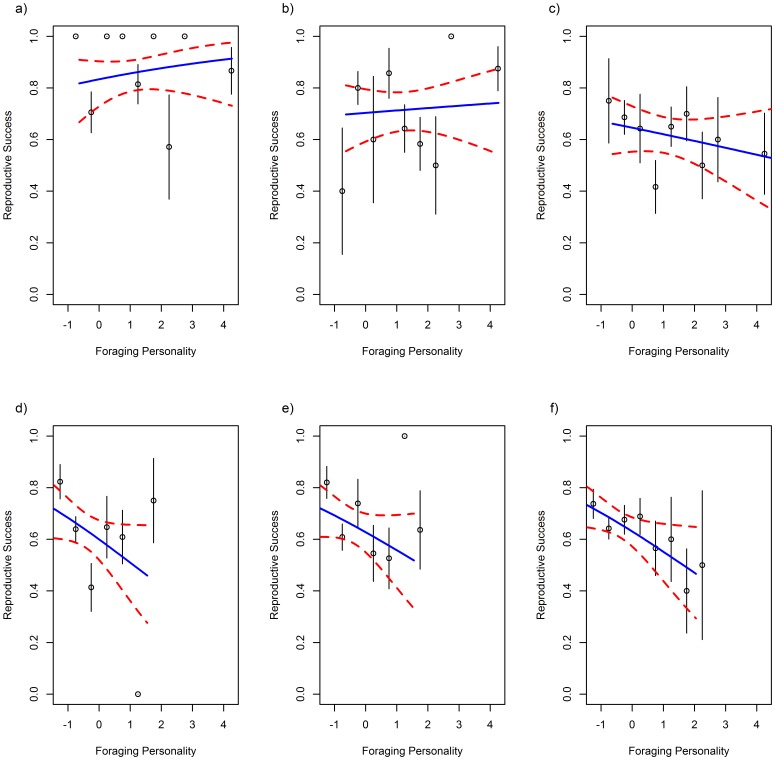
The relationship between foraging personality score and reproductive success across the last 23 years. a) Females Low SSTa years: = −0.60< SSTa <−0.36; b) Females Medium SSTa years: −0.36< SSTa <−0.15; c) Females High SSTa years: −0.15< SSTa <0.13; d) Males Low SSTa; e) Males Medium SSTa; f) Males High SSTa. Foraging personality, while being a continuous measure, is grouped here for plotting purposes only. As the raw data is formed of zeros and ones, plotting grouped means provides a much more informative plot. Points represent group means, with standard error bars. Model predictions are plotted in a solid line with 95% confidence intervals in dashed lines.

However, when considering the reproductive success over time, there was an interaction between sex, year quality and both boldness (χ^2^_4_ = 18.08; p = 0.001; Table 4) and foraging personality score (χ^2^_4_ = 15.32; p = 0.004; Table 4). Bold females had a higher fitness, which was particularly strong in good years of high SSTa (Figure 3a–c). This was coupled with evidence that in years of high SSTa, females with a low foraging personality, who forage nearer the colony, had a higher fitness but in low SSTa years, females foraging further from the colony had higher fitness (Figure 4a–c). In males, shy birds (Figure 3d–f) and those with a low foraging personality score always had higher fitness (Figure 4d–f), but these relationships were strongest in years of low SSTa.

## Discussion

We found that foraging behaviour is highly repeatable in black browed albatross and hence it can be consider as a personality trait. Furthermore, this trait and the widely considered personality trait of boldness correlate with aspects of reproductive success. We suggest these scores may form part of a behavioural syndrome, with bolder birds foraging on the shelf edge, closer to the breeding grounds, and this syndrome was particularly marked in males. These results are linked to fitness, with evidence of sex by personality interactions with year quality, indicative of food availability. Together, this indicates that selection may vary in its magnitude and direction between the sexes and personality measures, depending on environmental covariates, revealing the complex nature of personality and foraging in seabirds.

Environmental parameters, indicative of prey abundance, have been shown to interact with personality to produce fluctuating selection across years [43], [44]. As such the fitness benefit of different phenotypes change with the environmental conditions, resulting in varying selective pressures. The strength and direction of selection on personality was mediated by sex. In males, shyer individuals always had higher fitness, whereas this relationship was reversed in females, where bolder individuals have higher reproductive success. Previous studies have found sex mediated selection on personality and a meta-analysis suggests that bolder individuals have higher reproductive success, and this is particularly strong in males [49]. Our results however indicate that high boldness may be more adaptive to females, who have been shown to be subordinate to males in many species [50]. As such an increased boldness may help individuals compete for food [1], [51], [52] with the strength of this relationship being strongest in years of high quality, and perhaps high competition.

This mechanism is supported by evidence that low foraging personality scores are always advantageous in males, but in females, foraging near the colony is only supported in years of high quality. We suggest that females can only obtain sufficient food near the colony in high quality years and so in years of lower food availability, this strategy is less successful. Furthermore the quality of year influences the strength of the relationships reported, showing that in poor quality year, boldness has a weaker correlation with fitness in females but a stronger relationship in males. The positive relationship in females between boldness and fitness in high quality years may be linked to females foraging nearer the colony in these years, where boldness may be adaptive. However, the results for males suggest that bolder males do better in years of high food abundance, suggesting boldness does not simple predict competitive ability in males.

Demonstrating the selective pressures differ between the sexes and personality types raises questions regarding the causes of fitness differences. In this paper, we show that boldness correlates with the foraging habitat, as predicted by oceanographic features. Bolder birds forage in the shallow waters near the colony, where competition is predicted to be higher [53]. Whereas shy birds feed further from the colony, in deeper waters, and as competitive interactions are known to be costly in some species e.g. [54], [55], [56], and shyness is associated with a reduced propensity to take risks in other species [57], shy individuals may select foraging areas further from the colony, where competitive interactions may be reduced. Second, boldness has also been linked to exploration behaviour, such that bolder individuals explore more superficially [2], [58] and this may lead them to forage on the first available patch. As in our population the most productive foraging zones are located along the shelf edge [25] and in this species foraging in these areas is thought to be optimal [30], shy individuals may travel further to seek areas of reliable high quality, showing a risk adverse strategy or they locate these areas through more thorough exploration. Furthermore, recent work has shown that shyer individuals rely more on memory, as opposed to routine based searching [59], which may enable them to repeatedly locate highly profitable patches, further from the colony, at a lower cost than bolder birds. As black browed albatross have a highly varied diet and prey choice can be linked to their foraging habitat [25], these results suggest that personality measures may predict the diet of individuals and future work will use stable isotope analysis to examine potential individual dietary specialisation. Given that boldness is associated with decreased reproductive success in males but not females, there could be sexual segregation in prey choice which could mediate these differences.

We found no influence of boldness or foraging personality score on the association with fishing vessels in this population. Competition can be high around fishing boats [60] and although we predict this should favour bold birds, in this population there is a strong correlation between vessel presence and proximity to the colony, with boats located along the shelf edge (Electronic supplementary material; Appendix S3; Figure S1 in Appendix S3). Therefore if bolder individuals are able to compete for food close to the colony, they may not encounter fishing vessels. Future work, using a population where fishing activity occurs close to the colony could test whether bold individuals select to feed at vessels when there is no trade-off with distance travelled. Given that accidental by-catch of seabirds by long line fisheries is still of considerable conservation concern overall [61], a better understanding of individual variation in discard use would be extremely valuable. In particular, since attraction to fishing vessels causing mortality may have strong consequences for the population dynamics if a particular personality is affected [62], further work on personality effects on population dynamics would be very interesting.

This is one of only a handful of studies to report the existence of personality differences in seabirds [6], [10], [11]. Our PCA analyses show that consistency in foraging behaviour can be considered to be personality variation. While both measures of personality had a repeatability above 0.30, there were insufficient repeats to determine whether boldness was significantly repeatable. In a closely related species, the wandering albatross, boldness scores were highly repeatable when based on a large sample size [6]. It is important to address this problem in future work, and to encourage studies measuring the heritability of personality traits in seabirds, which would be integral to understanding how such variation is maintained in the population. Our results suggest that boldness and foraging personality score may be part of a behavioural syndrome and studies should continue to investigate syndromes and the presence of sex differences in occurrence or structure.

In summary, our results suggest that individual personality differences are important for seabird foraging behaviour and offspring survival. These data demonstrate that personality traits may be under fluctuating selection across years and emphasises the importance of sex specific behaviour in seabirds. It would be interesting to examine whether personality is correlated with individual adult survival and this will be possible in time. Furthermore, while understanding the heritability of these personality traits is essential to help explain their emergence and persistence in the population, this paper supports the link between personality differences, foraging behaviour and fitness in black browed albatross.

## Supporting Information

Appendix S1 **Foraging behaviour – comparisons between incubation and chick guarding.**
(DOCX)

Appendix S2 **Measuring boldness – a full ethogram.**
(DOCX)

Appendix S3 **Analysing fisheries data.** Figure S1, The spatial distribution of fishing vessels.
(DOCX)
