# Supplementary material for: Personality, Foraging and Fitness Consequences in a Long Lived Seabird
Source: PLoS One. 2014 Feb 4;9(2):e87269. doi: 10.1371/journal.pone.0087269 (PMC3913606; doi:10.1371/journal.pone.0087269)
Supplement: Appendix S1 — Foraging behaviour – comparisons between incubation and chick guarding. (DOCX) [file pone.0087269.s001.docx]

**Electronic Supplementary Information:**

**Personality, foraging and fitness consequences in a long lived seabird**

**Patrick and Weimerskirch**

**Appendix S1: Foraging behaviours**

Incubation and chick guarding

**Mean duration of trips (± SE)**

Chick guarding 50.64 ± 2.00 hours

Incubation 109.56 ± 9.10 hours

**Mean distance travelled (± SE)**

Chick guarding 372.11 ± 33.73 km

Incubation 1340.10 ± 134.57 km

The samples sizes were insufficient to examine the relationship between boldness and foraging during incubation. As the birds on eggs were late breeders in this study, it is not possibly to determine whether these differences in behaviours represent variation between incubation and chick rearing, or individual quality differences. Therefore to ensure our results were conservative, we excluded these data from foraging analyses.
