# Supplementary material for: Personality, Foraging and Fitness Consequences in a Long Lived Seabird
Source: PLoS One. 2014 Feb 4;9(2):e87269. doi: 10.1371/journal.pone.0087269 (PMC3913606; doi:10.1371/journal.pone.0087269)
Supplement: Appendix S2 — Measuring boldness – a full ethogram. (DOCX) [file pone.0087269.s002.docx]

**Electronic Supplementary Information:**

**Personality, foraging and fitness consequences in a long lived seabird**

**Patrick and Weimerskirch**

**Appendix S2: Measuring boldness: Full ethograrm**

Individuals were classified into one of three states at all times: Sitting, standing or on tarsus (define as resting on the tarsus but with the belly lifted from the nest). For these behaviours the duration, out of a maximum of 30 second, was reported. As birds rarely stood, the duration of time on tarsus was strongly negatively correlated with the duration of time sitting. Therefore we included only duration of time sitting in the final principal component analysis (PCA).

Events, recorded as a frequency, could occur during state. For each individual we recorded:

- Peck – contact between the bill and the ball
- Lunge – movement clearly towards ball, without contact
- Snap – opening and closing of the bill, not targeted at ball
- Vocalisation – any vocalisation
