# Supplementary material for: Personality, Foraging and Fitness Consequences in a Long Lived Seabird
Source: PLoS One. 2014 Feb 4;9(2):e87269. doi: 10.1371/journal.pone.0087269 (PMC3913606; doi:10.1371/journal.pone.0087269)
Supplement: Appendix S3 — Analysing fisheries data. Figure S1, The spatial distribution of fishing vessels. (DOCX) [file pone.0087269.s003.docx]

**Electronic Supplementary Information:**

**Personality, foraging and fitness consequences in a long lived seabird**

**Patrick and Weimerskirch**

**Appendix S3: Methods: Analysing fisheries data**

Fishing vessels discard unwanted catch and offal from processed fish. This creates an artificial food source for seabirds, and huge aggregations can be seen behind discarding vessels. The overlap with fishing vessels can be divided into different behaviours. There is a dramatic increase in number of birds once discards have been released, suggests the majority of individuals join the boat only to feed. Fishing locations were transmitted at various temporal intervals and when combined with line setting and hauling locations, the position of vessels could be estimated on average (± SE) every 37.25 ± 0.35 minutes and were used for analyse. In this study system there is a strong correlation between the location of vessels and the shelf edge. As such only birds which forage on the shelf edge overlap with fishing vessels [see also 1,2; Figure S1].

Figure S1: The relationship between depth and distance from the colony. The location of the shelf edge and the foraging area classification are highlighted. Black circles, of radius 19km, show the presence of fishing activity.


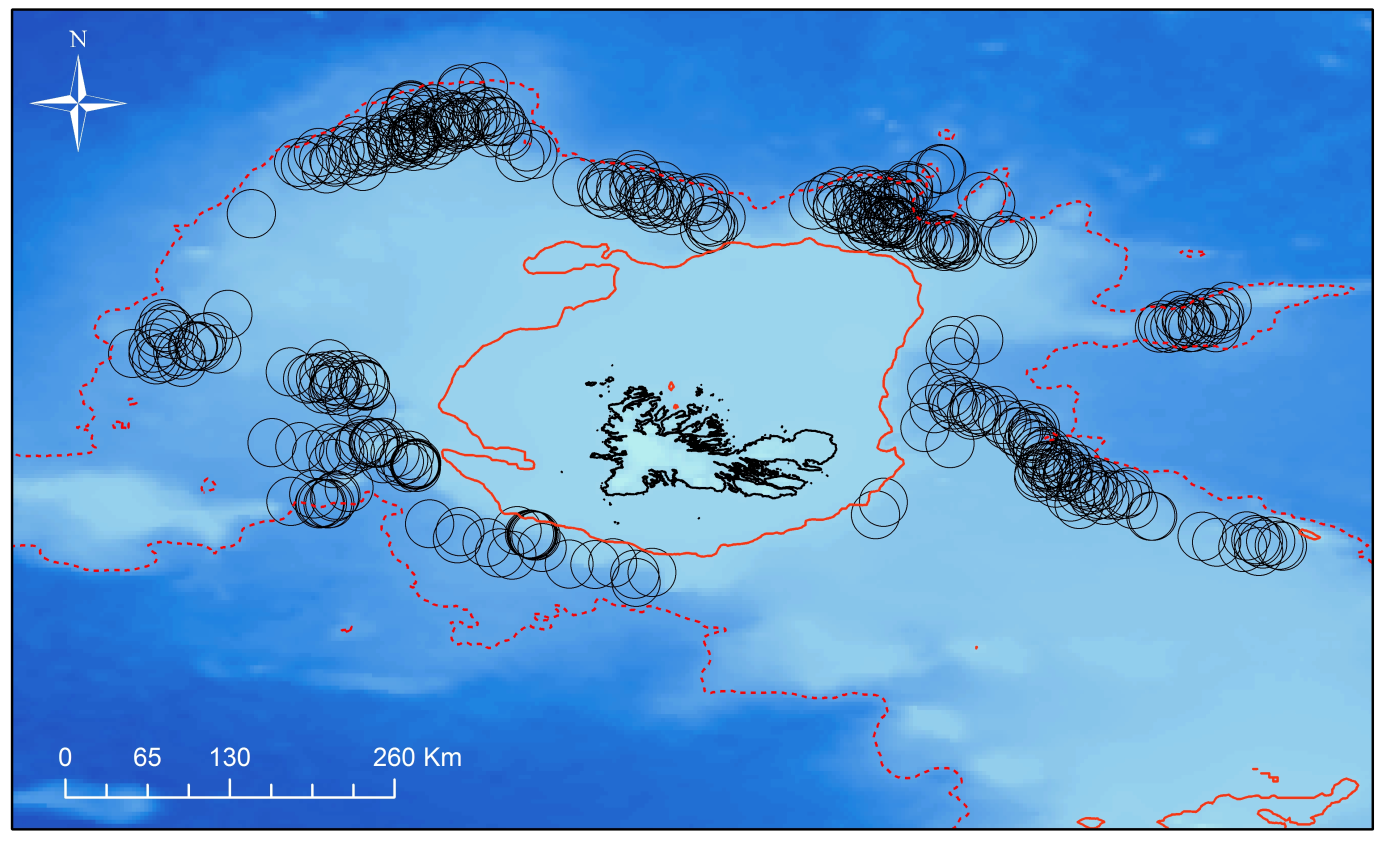


**References**

1. Cherel Y, Weimerskirch H, Duhamel G (1996) Interactions between longline vessels and seabirds in Kerguelen waters and a method to reduce seabird mortality. Biological Conservation 75: 63-70.

2. Weimerskirch H, Capdeville D, Duhamel G (2000) Factors affecting the number and mortality of seabirds attending trawlers and long-liners in the Kerguelen area. Polar Biol 23: 236-249.
